# Clinical profile of dengue in the elderly using surveillance data from two epidemics

**DOI:** 10.1590/0037-8682-0290-2021

**Published:** 2022-02-25

**Authors:** Yara Hahr Marques Hökerberg, Fernanda Kohn, Taís Suane de Souza, Sonia Regina Lambert Passos

**Affiliations:** 1 Fundação Oswaldo Cruz, Instituto Nacional de Infectologia Evandro Chagas, Laboratório de Epidemiologia Clínica, Rio de Janeiro, RJ, Brasil.; 2 Universidade Estácio de Sá, Faculdade de Medicina, Rio de Janeiro, RJ, Brasil.; 3 Fundação Oswaldo Cruz, Escola Nacional de Saúde Pública, Programa de Pós-Graduação em Epidemiologia em Saúde Pública, Rio de Janeiro, RJ, Brasil.

**Keywords:** Dengue, Elderly, Disease attributes, Public health surveillance

## Abstract

**Background:**

Population aging and mobility have increased the exposure of elderly individuals to dengue. This study evaluated the clinical features of dengue in the elderly during the epidemic (2008 and 2012) and interepidemic (2009 and 2010) periods.

**Methods:**

This cross-sectional study was based on dengue surveillance data from Rio de Janeiro, Brazil: 2008 (n=31,210), 2009‒2010 (n=2,884), and 2012 (n=30,773). The analysis was stratified by age group (<60 and ≥60 years).

**Results:**

Case-fatality rates were higher in the elderly. In 2008, elderly individuals were found to be more prone to hematuria and thrombocytopenia.

**Conclusions:**

These results can improve the understanding of dengue in elderly individuals who live in or travel to tropical regions.

Dengue in the Americas primarily affects adults^1^ ^,^ ^2^. Population aging has led to an increase in the proportion of elderly residents in tropical areas, increasing their vulnerability to dengue infection. The elderly are frailer and more prone to developing complications from diseases, which can be associated with physiological factors, immune alterations, comorbidities, or external factors, such as environmental conditions in the place of residence. Compared to younger individuals, elderly persons tend to remain hospitalized longer, and their comorbidities can aggravate the clinical condition of dengue^3^. Atypical presentations of dengue in the elderly can hinder the diagnosis of this arboviral infection^3^. The typically low frequency of mucosal bleeding in the elderly, in addition to physiological factors and frequent comorbidities in this age group, can be associated with the use of multiple medications^4^ ^,^ ^5^.

Early identification and timely treatment of dengue cases with the potential to evolve with severity are necessary to reduce morbidity and mortality from this disease^6^. However, although elderly individuals represent the fastest growing population group worldwide and are potentially more susceptible to dengue infection, there are few specific studies on the clinical profile of dengue in this population. Most studies have assessed small hospital samples in Asia and the Americas without identifying the viral serotype^7^. Few existing studies have employed widely varying methods, which affects the comparability of their results.

In Brazil, dengue is a mandatorily reported disease, the cases of which are periodically recorded in the Information System on Diseases of Notification (SINAN^8^), which allows a dynamic diagnosis of the occurrence of events in the population, helping to draft appropriate public health strategies.

Two major dengue epidemics occurred in the city of Rio de Janeiro in 2008 and 2012, and the predominant circulating serotypes were DENV-2 and DENV-4, respectively. Dengue classification in these two epidemics was based on national manuals in force at the time. In 2008, dengue was classified by the WHO^9^ as dengue fever (DF) and dengue hemorrhagic fever (DHF); Brazil^8^ included a new category, dengue with complications (DC), for cases that evolved with severity or caused mortality but failed to meet the clinical and laboratory criteria for DHF. In the 2012 epidemic, although the new WHO classification, namely DF with or without warning signs and severe dengue, was already in force (2009), Brazil still used the classification from the national manual^8^. 

However, none of the manuals presented specificities related to clinical management in the elderly, probably because of the small number of studies in this population subgroup, particularly in the Americas. The current study aimed to assess the clinical features of dengue in the elderly in two epidemics in the city of Rio de Janeiro in 2008 and 2012, when the main circulating serotypes were DENV-2 and DENV-4, respectively. Moreover, we compared the potential clinical differences or similarities observed among elderly patients during the 2008 epidemic with those during the interepidemic period (2009-2010).

A cross-sectional descriptive study was conducted according to the guideline REporting studies Conducted using Observational Routinely collected health Data - RECORD^10^, based on the available information in the Information System on Diseases of Notification (SINAN). Rio de Janeiro is the second largest Brazilian city in both demographic and economic terms, with a population of 6,320,446 in 2010, located on the seacoast in the southeast of the country with great tourist potential, having hosted several major international events. 

This study included dengue cases reported to SINAN in the resident population of the city of Rio de Janeiro. Confirmatory criteria for dengue were those of the Brazilian Ministry of Health^8^, namely viral RNA detection by reverse transcriptase polymerase chain reaction (RT-PCR) and capture enzyme-linked immunosorbent assay (ELISA) for IgM antibodies or IgM or IgG seroconversion (ELISA) in paired samples. In the absence of laboratory confirmation, a clinical-epidemiological criterion was applied, defined as a clinical picture consistent with dengue, an epidemiological link to a laboratory-confirmed dengue case, and the absence of other differential diagnoses consistent with the patient´s age group^8^. To avoid selection bias, we opted to include cases confirmed by the clinical-epidemiological criterion because, during epidemics, most cases with laboratory confirmation were those evolving to severe forms, such as DHF. The exclusion criteria were patients without information on age, place of residence, or clinical variables. 

In 2008 and 2012, 124,037 and 178,805 dengue cases were reported in the city of Rio de Janeiro, respectively. Of the 302,842 reports in this sample, 51,172 were ruled out as dengue, 809 had no information on residence, 154 resided outside the city of Rio de Janeiro, and 65 individuals were 100 years or older, totaling 250,642 (120,381 in 2008 and 130,261 in 2012). Of these, 61,983 reports had complete information on clinical characteristics (31,210 in 2008 and 30,773 in 2012).

The following clinical and sociodemographic variables were analyzed: age (less than 60 vs. 60 years or older), sex, clinical classification (dengue fever, dengue with complications, and dengue hemorrhagic fever) according to the prevailing classification at time^8^, and evolution (cure/death). For cases classified as DC or DHF, we evaluated the presence of hemorrhagic manifestations, signs of plasma leakage, and platelet count nadir (per mm3). 

The analyses were stratified according to the age group. Due to the descriptive cross-sectional design and sample size, the association between qualitative variables and the clinical form (dengue fever, dengue with complications, and dengue hemorrhagic fever) was based on the comparison of absolute and relative frequencies, considering the level of statistical significance (p<0.01), or differences of 10 percentage points for comparisons in strata with a small number of cases. Platelet count is described in values of median and interquartile range. Single and multiple regression analyses were not performed because clinical predictors, such as bleeding and plasma leakage, are part of the outcome definition criterion (clinical form), which would cause a common source bias. Statistical analyses were performed using the SPSS version 16.

The study used the municipal database of SINAN, obtained from the Rio de Janeiro Municipal Health Department, following approval by the Institutional Review Boards of the National School of Public Health Sergio Arouca of the Oswaldo Cruz Foundation (CAAE 58814516.2.0000.5240) and Rio de Janeiro Municipal Health Department (CAAE 58814516.2.3001.5279).

The current study included 64,867 cases with complete information on clinical characteristics, 31,210 reports from 2008, 2,884 from 2009 to 2010, and 30,773 from 2012, of which 2,328 (7.5%), 213 (7.4%), and 2,788 (9.1%) were elderly individuals, respectively. In the epidemic years, there were more elderly women. In both the epidemic and interepidemic periods, elderly patients had less schooling on average than younger patients (p < 0.001). The criteria used for dengue diagnosis were mostly clinical-epidemiological in the epidemic in 2008 and laboratory in 2012, and the elderly had a higher proportion of laboratory confirmation (p <0.001) (Table 1). 

The epidemic in 2008 was more severe, as evidenced by the higher proportion of cases of DC, DHF, and deaths (Table 1). In 2008, of the 902 cases classified as DHF, 741 had information on the level of severity, 90 of which presented with dengue shock syndrome (< 60 years: n=79, 11.5%; ≥ 60 years: n=11, 21.2%). In 2012, only 94 cases evolved to DHF, 16 of 81 with information on level of severity evolved to dengue shock syndrome (< 60: n=12, 16.4%; ≥ 60: n=4, 50%); in the interepidemic period, they represented 7 of 85 DHF cases (< 60: n=5, 6.3%; ≥ 60: n=2, 33.3%) (data not shown).

Although the proportion of cases with greater severity was similar across age groups, case fatality in the elderly was higher during both epidemics, approximately seven-fold in 2008 and five-fold in 2012 when compared to younger individuals (Table 1).

**TABLE 1:** Sample’s description (N=64,867).

	2008 (n=31,210)					2009-2010 (n=2,884)					2012 (n=30,773)				
Variables	< 60 years		≥ 60 years		P^a^	< 60 years		≥ 60 years		P^a^	< 60 years		≥ 60 years		P^a^
	n	(%)	n	(%)		n	(%)	n	(%)		n	(%)	n	(%)	
**Sex**					*					***					*
Female	15556	(53.9)	1.400	(60.1)		1247	(46.7)	116	(54.5)		14973	(53.5)	1668	(59.8)	
Male	13326	(46.1)	928	(39.9)		1423	(53.3)	97	(45.5)		13012	(46.5)	1120	(40.2)	
**Color**					*					**					*
White	4324	(48.6)	412	(68.3)		472	(55.1)	45	(73.8)		5016	(44.8)	538	(53.8)	
Other	4566	(51.4)	191	(31.7)		385	(44.9)	16	(26.2)		6178	(55.2)	462	(46.2)	
**Education**					b*					**					b*
Primary	4289	(49.9)	303	(88.1)		307	(38.2)	17	(58.6)		2713	(40.8)	221	(61.6)	
Secondary or +	671	(7.8)	32	(9.3)		198	(24.7)	11	(37.9)		2289	(34.4)	138	(38.4)	
Not applicable^c^	3641	(42.3)	9	(2.6)		298	(37.1)	1	(3.4)		1653	(24.8)	-	-	
**Diagnostic**					*					*					*
Laboratory	9858	(34.1)	1.035	(44.5)		981	(37,1)	109	(52.4)		19638	(70.2)	2.084	(74.7)	
Clinical-Epidemiological	19024	(65.9)	1293	(55.5)		1661	(62.9)	100	(47.8)		8347	(29.8)	704	(25.3)	
**Evolution**					b^*^					b**					b*
Cure	21272	(99.5)	1.622	(96.7)		1777	(99.5)	127	(96.2)		25127	(99.9)	2.476	(99.4)	
Death dengue	101	(0.5)	55	(3.3)		8	(0.4)	3	(2.3)		22	(0.1)	12	(0.5)	
Death others	1	(0.0)	1	(0.0)		1	(0.1)	2	(1.5)		2	(0.0)	4	(0.2)	
**Classification**															
Dengue fever	20423	(70.8)	1.607	(69.1)		2161	(80.9)	167	(78.4)		27478	(98.4)	2.726	(98.0)	
Dengue with complication	7594	(26.3)	660	(28.4)		422	(15.8)	39	(18.3)		371	(1.3)	46	(1.7)	
DHF^d^	844	(2.9)	58	(2.5)		88	(3.3)	7	(3.3)		85	(0.3)	9	(0.3)	

^a^ P-value of χ2 Pearson: *p< 0.001, **p<0.01, ***p<0.05; ^b^P-value of Fisher’s test: *p<0.001, **p<0.01; ^c^Children; ^d^DHF: Dengue hemorrhagic fever.

In 2008, elderly patients classified as having dengue with complications showed a higher proportion of petechiae (p=0.012) and hematuria (p<0.001), whereas mucosal bleeding was less evident. In both age groups, plasma leakage was more frequent than bleeding, with hemoconcentration as the main sign, followed by cavitary effusions, which was less frequent in individuals 60 years or older. The median platelet nadir did not differ between age groups (Table 2 and Table 3).

**TABLE 2:** Signs of Dengue with Complications (DC) and Dengue Hemorrhagic Fever (DHF).

	2008 (n=9,156)							2009-2010 (n=556)						
		DC			DHF				DC			DHF		
Variables	n^a^	< 60	≥ 60		< 60	≥ 60		n^a^	< 60	≥ 60		< 60	≥ 60	
		n (%)	n (%)	P^b^	n (%)	n (%)	P^b^		n (%)	n (%)	P^b^	n (%)	n (%)	P^b^
Hemorrhages	8224	2452 (36.3)	169 (30.0)	**	803 (95.8)	56 (96.6)	^c^	515	164 (42.7)	14 (37.8)		86 (98.9)	7 (100)	
Epistaxis	3531	683 (27.0)	29 (16.9)	**	188 (24.3)	9 (17.0)		256	42 (26.9)	2 (15.4)		20 (24.7)	1 (16.7)	
Gums	3516	625 (24.8)	26 (15.2)	**	179 (23.1)	10 (19.2)		257	35 (22.3)	3 (23.1)		20 (24.7)	-	
Metrorrhagia	3485	255 (10.2)	5 (3.0)	**	69 (9.0)	1 (1.9)	^c^	253	9 (5.8)	1 (7.7)		2 (2.5)	-	
Petechiae	3545	877 (34.5)	76 (43.9)	***	370 (47.7)	31 (58.5)		261	75 (47.8)	9 (64.3)		54 (64.3)	2 (33.3)	
Hematuria	3473	93 (3.7)	22 (12.8)	*	33 (4.3)	4 (7.5)	^c^	254	21 (13.6)	2 (15.4)		4 (4.9)	2 (33.3)	
Gastrointestinal	3493	433 (17.3)	32 (18.9)		148 (19.3)	10 (18.9)		254	30 (19.5)	4 (30.8)		12 (14.8)	1 (16.7)	
Tourniquet test +	3041	186 (8.6)	17 (11.4)		95 (13.8)	9 (19.1)		231	15 (10.9)	-		15 (19.5)	2 (40.0)	
Plasma leakage	7957	3715 (56.7)	223 (42.3)	*	794 (96.6)	54 (96.4)		464	138 (40.9)	10 (30.3)			84 (96.6)	7 (100)
*Evidenced by*				^c^*			^c^							
Hemoconcentration	3599	2875 (77.4)	202 (90.6)		482 (60.7)	40 (74.1)		180	104 (75.4)	10(100)			62 (73.8)	4 (57.1)
Cavitary effusions	1068	773 (20.8)	16 (7.2)		268 (33.8)	11 (20.4)		54	31 (22.5)	-			20 (23.8)	3 (42.9)
Hypoproteinemia	119	67 (1.8)	5 (2.2)		44 (5.5)	3 (5.6)		5	3 (2.2)	-			2 (2.4)	-
Nadir Platelet (*1000/mm^3^)^d^	8462	34 (21-52)	29 (19-45)		25 (16-39)	20 (13-37)		93	43.5 (24-89)	43.5 (23-78)			28.5 (15-49.2)	33 (30-44)

^a^ Total valid cases; ^b^P-value of Pearson’s χ2 test: *p< 0.001, **p<0.01, ***p<0.05; ^c^P-value of Fisher’s test: *p<0.001, **p<0.01; ^d^median (interquartile interval).

**TABLE 3:** Characteristics of Dengue with Complications (DC) and Dengue Hemorrhagic Fever (DHF), 2012 (n=511).

Variables	n^a^	DC^b^		DHF^b^	
		< 60	≥ 60	< 60	≥ 60
		n (%)	n (%)	n (%)	n (%)
Hemorrhages (yes)	451	152 (48.6)	18 (40.9)	80 (94.1)	9 (100)
Epistaxis	245	31 (20.8)	1 (5.9)	20 (27.8)	1 (14.3)
Bleeding gums	245	34 (22.8)	2 (11.8)	17 (23.6)	-
Metrorrhagia	244	15 (10.1)	-	9 (12.5)	-
Petechiae	248	61 (41.5)	4 (23.5)	29 (38.2)	3 (37.5)
Hematuria	243	18 (12.2)	2 (11.8)	5 (7.0)	1 (14.3)
Gastrointestinal	244	27 (18.2)	4 (23.5)	11 (15.3)	1 (14.3)
Tourniquet test +^c^	232	24 (17.5)	10 (58.8)	20 (28.2)	3 (42.9)
Plasma leakage (yes)	423	139 (47.3)	22 (53.7)	65 (81.2)	7 (87.5)
*Evidenced by*					
Hemoconcentration	181	112 (80.6)	19 (86.4)	45 (69.2)	5 (71.4)
Cavitary effusions	50	27 (19.4)	3 (13.6)	18 (27.7)	2 (28.6)
Hypoproteinemia	2	-	-	2 (3.1)	-
Nadir Platelet *1000/mm^3 c^	403	28 (16-69)	30 (15-58)	33 (18-53)	

^a^Total valid cases; ^b^Non-significant statistical differences Pearson’s chi-square or Fisher’s exact test (p>0.05); ^c^Fisher’s exact test p<0.01 in DC strata.

Our study showed that case fatality from dengue was higher in the elderly, especially in 2008, the year with the most severe epidemic, probably due to the circulation of the DENV-2 serotype^11^. In cases that developed complications in 2008, the elderly had a higher proportion of petechiae and hematuria, while plasma leakage was less frequent.

Higher fatality from dengue in the elderly has also been a frequent finding in other studies^6^ ^,^ ^12^ ^-^ ^14^ and appears to be related to the senescent immune system and common comorbidities in this population group. In the presence of dengue infection, in vitro experimental studies have demonstrated that both newborns and the elderly exhibit physiological immunosuppression, resulting in a lower inflammatory response and control of the infection^15^.

Comorbidities such as hypertension, diabetes, and cardiovascular, renal, and chronic obstructive pulmonary diseases^3^ ^,^ ^6^ ^,^ ^14^ are commonly associated with polypharmacy, which can influence the correct diagnosis and clinical management of the disease, thus leading to more severe evolution and death^7^ ^,^ ^13^.

The epidemic in 2008 had a higher proportion of severe cases, possibly due to the predominant circulation of the DENV-2 serotype. A meta-analysis of 20 studies showed that infection with this serotype increased the odds of developing dengue shock syndrome by 66%^11^.

Our results suggest that the distribution of complications and DHF in the elderly was similar to that in younger patients, contrary to other studies showing a higher frequency of DHF in the elderly^3^ ^,^ ^6^ ^,^ ^12^. Although the frequency of bleeding was similar between the age groups, our findings showed that the elderly who developed complications in 2008 had higher proportions of petechiae and hematuria. 

In both epidemics, hemoconcentration was the principal manifestation of plasma leakage, with no difference between the age groups. Cavitary effusions were less frequent in elderly patients (especially in 2008) than in younger patients, contrary to the findings in the literature^12^ ^,^ ^14^. However, most studies on dengue in the elderly have used hospital samples and may present a selection bias. In addition, elderly individuals tend to present with dehydration and lower fluid intake during an illness, which can combine with immune senescence to clinically mask plasma leakage^15^.

To our knowledge, this is the first study to assess the clinical features of dengue in the elderly in a large population-based sample, considering the epidemic and interepidemic periods. Most of the studies previously published have included hospital samples with fewer cases from the Americas^7^. Meanwhile, our study’s main limitations were the high proportion of missing values on clinical variables and the criteria used for dengue definition. Our study sample was older than that of the entire database. While the clinical-epidemiological criterion could lead to a classification bias, the exclusion of cases without laboratory confirmation could produce a selection bias, particularly in epidemic periods when resources are depleted. In addition, the high positive predictive value for dengue diagnosis associated with a higher incidence tends to minimize classification errors from the case definition criteria.

We conclude that in both epidemic and interepidemic years, older dengue patients were at larger risk of mortality, but presented with less evidence of plasma leakage. Therefore, physicians should be alert when performing fluid replacement in these cases, because capillary permeability may be altered, even if clinical evidence is lacking. Another finding in this study was the higher frequency of petechiae and hematuria in the elderly in 2008, which was probably related to the DENV-2 serotype^11^. New prospective studies with population-based samples would improve our understanding of the clinical evolution of dengue in the elderly, with special attention paid to elucidating possible differences in the mechanisms and clinical manifestations related to plasma leakage.
